# Path to future markets: Method for quantifying long-duration energy storage competitiveness

**DOI:** 10.1016/j.isci.2026.115751

**Published:** 2026-04-15

**Authors:** Farzan ZareAfifi, Zabir Mahmud, Sarah Kurtz

**Affiliations:** 1 School of Engineering, University of California, Merced, Merced, CA 95343, USA

**Keywords:** applied sciences, energy storage, materials application

## Abstract

Energy storage technologies with a range of durations are essential for addressing solar and wind generation variability in renewable-energy-driven grids. This study defines long-duration energy storage (LDES) as a system where the energy reservoir can be scaled independently of the power conversion hardware and introduces a modeling method to quantify LDES competitiveness relative to lithium-ion batteries (LiBs), today’s dominant grid-scale storage. To demonstrate the model, this study examines California’s solar-dominated grid to identify the LDES reservoir size that will be most cost-competitive by balancing both power and energy cost competitiveness. Further model demonstration quantifies how scaling up the energy reservoir size relative to the power rating can enable LDES to compete even at efficiencies as low as 30% for strict carbon-emissions scenarios, playing a crucial role in maintaining grid resiliency. While LDES may support reliability by providing reserve capacity, this model minimizes electricity delivery cost to a predefined load.

## Highlights

• Market-share method defines power and energy cost targets for LDES market entry
• 8-h LDES balances power and energy cost competitiveness in solar-dominated grid
• 100-h LDES, even at low RTEs, gains a strong value under zero-carbon constraints
• Method adapts to diverse grid policies and scenarios, such as wind or resilience-driven

## Graphical abstract

**Figure:**
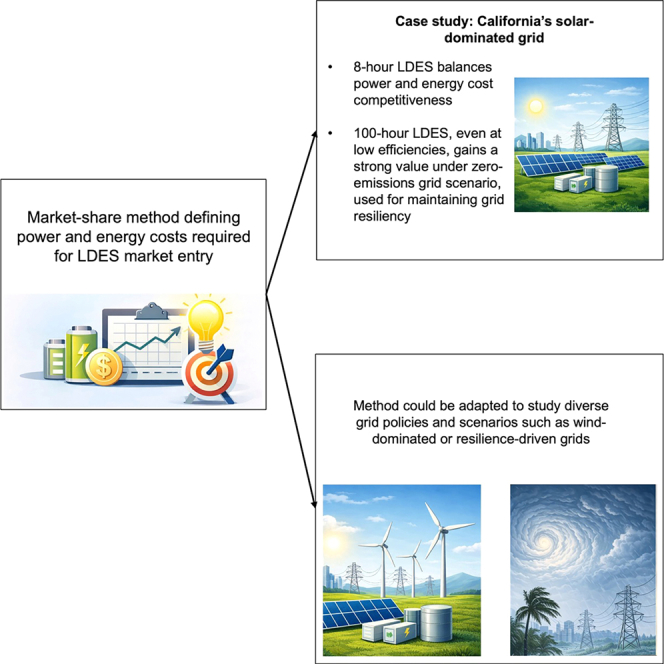

## Introduction

Addressing the variability of wind and solar power is becoming increasingly critical as we transition toward renewable-energy-driven grids. Coupling these variable energy sources with storage technologies, following the recent significant reductions in lithium-ion battery (LiB) costs,^1^ ensures reliable operation and dispatchability, thereby supporting the increased adoption of renewable power generation.^2^ For example, the capacity of LiBs in California has grown significantly in recent years, reaching 13 GW as of September 2024.^3^ This rapid expansion helps store the excess solar generation produced during the day for use after sunset when solar electricity becomes unavailable.

LiBs are well-suited for instantaneous and short-duration energy balancing. However, longer-duration energy storage (LDES) technologies with lower-cost energy reservoirs may provide a lower-cost approach to complementing solar and wind electricity generation.^4^ LDES may play a key role in both cost-optimized firm energy systems and in mitigating power generation shortages. Yang et al.^5^ emphasized that installing LDES alongside short-duration energy storage can significantly reduce the overall cost of a firm photovoltaic (PV) system compared to using short-duration storage alone.

Table 1 summarizes the focus areas of key prior studies on LDES value and competitiveness as a basis for evaluating how this study builds upon, advances, or complements that literature. As highlighted in the table, prior studies have primarily focused on evaluating the system-level value of LDES under specific modeling assumptions, often with an emphasis on total cost reductions, avoided curtailments, or firm capacity contributions.

**Table 1:** Summary of key contributions from prior literature and additional value added by this study

Study	Contributions	Complementary value added by this work
Sepulveda et al.^6^	developed a method to evaluate the system-level value of LDES, such as total system cost reduction, based on three key performance metrics: energy cost, power cost, and round-trip efficiency.	introduces a market-share-based method that shifts the focus from system-level metrics to identifying cost thresholds required for LDES to capture defined market shares.
Albertus et al.^7^	reviewed different LDES technologies and application domains with the emphasis on the importance of achieving low energy-related costs (e.g., $1–10/kWh) and moderate round-trip efficiency (e.g., >60%) to enable cost-competitive deployment for durations exceeding 50 h.	introduces a technology-agnostic framework that identifies performance metrics, rather than specific technologies, required for market entry under a given grid configuration and market design.
Jenkins and Sepulveda^8^	evaluated LDES system value based on key technical parameters: energy capacity cost, charge/discharge power cost, and charge/discharge efficiency.	introduces a market-share-based method that shifts the focus from system-level metrics to identifying cost thresholds required for LDES to capture defined market shares.
Selanniemi et al.^9^	analyzed how carbon pricing and other decarbonization policies influence the economic viability and system value of LDES technologies. Highlighted that current market structures often undervalue LDES and that policy interventions are critical to unlock their full value.	introduces a flexible framework that can be applied across different policy environments and grid configurations, rather than evaluating a limited set of policy scenarios.
Denholm et al.^10^	quantified key value streams for LDES and emphasized how the value of LDES is highly dependent on system context and deployment scale.	provides a market-share-oriented valuation approach that can be applied consistently across different system contexts to quantify LDES competitiveness.
Hargreaves and Jones^11^	used a U.S. capacity optimization model to show that deeply decarbonized power systems require diverse resource portfolios and that LDES must meet aggressive cost targets to contribute beyond diurnal balancing.	introduces a generalized method that identifies performance requirements for market entry, rather than focusing on a single grid or market condition.
Liu et al.^12^	used a techno-economic optimization framework to show that coordinated portfolios of short-duration storage and LDES outperform single-technology solutions, depending on reliability targets and cost assumptions.	identifies lithium-ion batteries as the primary competitors to LDES and quantifies relative cost and performance thresholds required for LDES adoption under different grid conditions.

Although LDES may play a key role in cost-optimized firm energy systems, its adoption levels depend on cost assumptions when optimizing the total cost of electric grid systems. For example, Ziegler et al.^13^ demonstrated that when storage energy-capacity costs are high, storage adoption is reduced by relying on oversizing renewable generation, accepting higher curtailments, to meet demand. This raises the question of whether current LDES technologies are more likely to play a key role or whether oversizing renewable generation may provide a lower cost. To answer this question, Sepulveda et al.^6^ explored various LDES technologies, including electrochemical, chemical (e.g., hydrogen), thermal, and mechanical storage solutions, and found that displacing firm generation with LDES technologies, particularly in northern regions with high seasonal heating demand, remains challenging given the current performance parameters of known technologies.

Similarly, Albertus et al.^7^ highlighted the critical need to reduce LDES costs significantly for competitiveness. Specifically, they suggested that energy storage subsystem costs must fall below $10/kWh for durations exceeding 50 h. Jenkins et al.^8^ identified charging and discharging efficiencies, alongside energy costs, as critical performance metrics for determining the competitiveness of LDES technologies. They argued that for LDES technologies to provide substantial system value and savings in electricity costs for decarbonized grids, energy capacity costs must fall as low as $1–10/kWh, and discharge efficiencies must exceed 60%. ZareAfifi et al.^14^ reviewed current LDES technologies, including metal-air and flow batteries, and found that they typically have energy costs far above this threshold. With respect to efficiency, Koohi-Fayegh and Rosen^15^ reported that electrochemical LDES technologies, such as flow batteries, can reach round-trip efficiency (RTE) values of 70%–80%, while others, such as hydrogen-based systems, exhibit lower efficiencies (30%–45%), as illustrated by Staffell et al.^16^ Collectively, these studies indicate that substantial improvements, epecially in energy-related costs, are required before many LDES technologies become competitive with lithium-ion alternatives.

Besides the metrics mentioned by Albertus et al.^7^ and Jenkins et al.,^8^ the successful deployment of LDES will also depend heavily on supportive policy and market environment. Selanniemi et al.^9^ argued that in the absence of such policies, current markets may undervalue LDES, limiting investment and deployment. As an example of such a policy, they highlighted that LDES becomes increasingly valuable in power systems subject to decarbonization constraints and under strict emission reduction targets. They highlighted that under such binding carbon caps, the operational and system-level benefits of LDES, such as avoiding renewable curtailments, supporting grid resiliency, and displacing peaker plants, are more fully recognized and monetized.

Studies such as Jenkins et al.,^8^ published in 2021, and Selanniemi et al.^9^ offer valuable insights into the important market design and performance metrics LDES technologies need to achieve to be competitive and provide system-wide value. However, as mentioned, the cost of LiBs has decreased significantly in recent years,^1^ and their adoption has accelerated rapidly. Schmidt et al.^17^ highlighted that LiBs are expected to dominate most stationary storage applications beyond 2030, primarily due to their rapid cost reductions. Denholm et al.^10^ further argued that for LDES to achieve cost parity with LiBs, advancements such as longer operational lifetimes and lower energy-related costs will be essential, particularly given the potential for higher power-related costs in many LDES technologies. They also emphasized the importance of continued support to facilitate early deployment, enabling cost reductions through learning and innovation. Consequently, unless alternative storage technologies can achieve substantial performance improvements that keep pace with LiB cost declines and in the absence of supportive policies, their widespread adoption remains uncertain. For example, even when a solicitation was intended for LDES, there have been times when LiB projects have been selected over LDES.^18^ This raises a critical question: To what extent and under what market conditions must LDES costs at a given performance level be reduced to begin capturing market share in an energy storage landscape increasingly dominated by LiBs and what size energy reservoir will be most competitive?

This latter question, which may be restated as “If I develop an energy storage product with a larger energy reservoir, how much higher price may I charge while capturing a similar market share?,” was a key motivator for this study. Traditional capacity optimization modeling does not give a direct answer to this question (Table 1). So, for this paper, we developed a method, as described in the STAR Methods Section. We demonstrate the value of this method by quantifying the competitiveness for each energy reservoir size as a function of the LDES efficiency, time frame, and carbon-emissions restrictions as a case study analysis. Thus, this study introduces a market-share-based method that identifies the cost thresholds at which LDES technologies, with specific design characteristics (e.g., RTE and energy reservoir size), can capture a defined share of the storage market. The proposed method can be adapted for analyzing different grid configurations, depending on where an LDES product is being marketed.

## Results and discussion

This section applies our cost-target method to the example case of California’s solar-dominated grid, analyzing the LDES costs required to achieve 1%, 10%, and 25% market share with respect to variations in the LDES energy reservoir size (measured in hours) and LDES RTE under California’s grid assumptions. Table 2 summarizes the analyzed LDES and grid modeling parameters and the corresponding ranges for which the cost target approach was conducted in this case study. Parameters in this table were evaluated at discrete values, shown by datapoints in the figures in this section. We evaluate LDES power and energy penetration levels of 1%, 10%, and 25% to capture a range of adoption stages, from initial market entry to more substantial deployment. As shown in this section, these penetration levels exhibit distinctly different cost-target behavior, particularly for LDES technologies with larger energy reservoir sizes (e.g., 100-h), providing useful insights into how cost competitiveness evolves with increasing market share. Regarding the carbon emissions scenario, please see the supplemental materials for more details. For three representative LDES (30% 100-h, 70% 100-h, and 80% 8-h), we further explored the cost targets for four target years and two carbon-emissions scenarios. It is important to note that an increase in power and energy cost targets indicates that LDES can enter the market at higher costs, signifying greater economic attractiveness and competitiveness. The section concludes with a discussion of the significance and applicability of the findings.

**Table 2:** Ranges of LDES and grid modeling parameters studied

LDES/grid modeling parameter	Round-trip efficiency (%)	Energy reservoir size (hours)	Modeled years	Carbon emissions scenarios	Penetration levels (%)
Studied range	min: 30 max: 85	min: 5 max: 100	2030, 2035, 2040, and 2045	“CA target” vs. “zero emissions”	1, 10, and 25

### Effect of long-duration energy storage reservoir size

To better understand the impact of energy storage reservoir size, consider the following thought experiment before reviewing the results. If the LDES energy reservoir size is small, more LDES units, including power conversion hardware, are required to generate the electricity needed. This leads to *power* overcapacity, causing the *power* cost target to decrease, meaning that LDES power becomes less valuable. Conversely, scaling up the energy reservoir size ensures sufficient *energy* availability, but a fixed amount of *power* conversion hardware is still required. If the energy reservoir is oversized relative to the need, it results in *energy* overcapacity, causing the *energy* cost target to decrease, meaning that *energy* storage capacity becomes less valuable.

**Figure 1:**
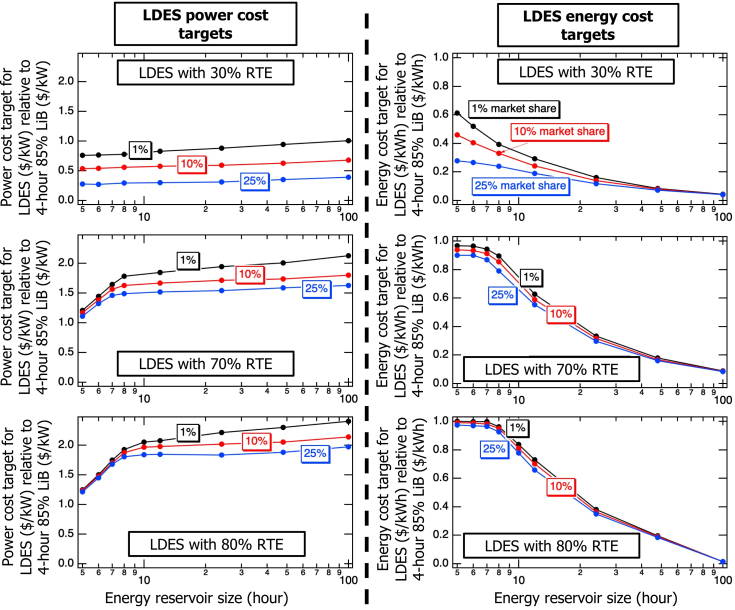
Cost ratios for LDES to reach penetration levels of 1%, 10%, and 25% according to the storage power (left) and energy (right) ratings for 30%(top), 70% (middle), and 80% (bottom) LDES as a function of energy reservoir size assuming the “CA target” carbon emissions scenario for the year 2045

Figure 1 illustrates the power and energy cost ratio targets for the three market penetration levels of 1%, 10%, and 25% as a function of LDES energy reservoir size for assumed 30%, 70%, and 80% RTEs. As discussed in the previous paragraph, the power cost targets increase monotonically, and the energy cost targets decrease monotonically as the energy reservoir size scales up, as expected; however, Figure 1 reveals an unexpected inflection point for the LDES with 70% and 80% RTEs. The graphs in Figure 1 indicate that 8-h LDES with 70% and 80% RTEs, which represent a realistic RTE range for this energy reservoir size,^19^ serves as a critical balance point. For LDES energy reservoirs exceeding 8 h, there is a pronounced decrease in energy cost ratio targets (with power cost ratio targets almost constant), while for LDES energy reservoirs smaller than 8 h, there is a sharp and approximately linear drop in power cost ratio targets (with energy cost ratio targets almost constant). This suggests that if only one type of LDES is used, 8-h LDES may serve as an optimal balance point in California’s solar-dominated grid, maintaining both power and energy values relatively high across the range of LDES energy reservoir sizes. However, as LDES penetration increases, this transition point shifts toward smaller energy reservoir sizes. For instance, at 25% penetration of 70%-efficient LDES, the optimal balance point occurs at approximately 7-h LDES energy reservoir size, as shown in Figure 1. This analysis highlights the tradeoff between LDES energy reservoir size and power and energy cost targets, emphasizing the importance of optimizing reservoir sizing for economic competitiveness.

### Effect of long-duration energy storage on round-trip efficiency

**Figure 2:**
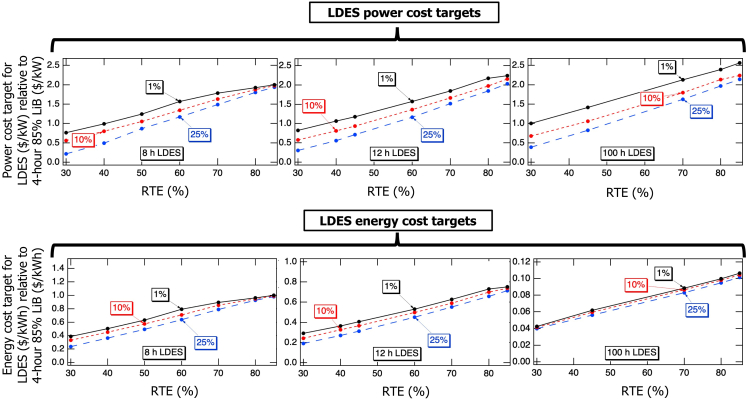
Cost ratio targets for LDES capturing 1%, 10%, or 25% of the storage market as a function of RTE for energy reservoir sizes of 8- (left), 12- (middle), and 100-h (right) for the “CA target” carbon emissions scenario for the year 2045 The y axes have been scaled to compare equal costs per kW for both the upper and lower graphs.

Figure 2 presents the power and energy cost targets for 8-, 12-, and 100-h LDES energy reservoir size relative to the defined LiB (see STAR Methods) to achieve 1%, 10%, and 25% market share by 2045 as a function of RTE. As expected, higher LDES RTE allows entry into the market at higher power and energy costs, indicating an increase in LDES attractiveness. For example, reducing RTE from 85% to 30% for 100-h LDES results in a drop of the power cost target by more than a factor of two, ultimately requiring that the cost of a 30% 100-h LDES be equal to or lower than the cost of a LiB with a similar power rating to achieve even 1% penetration. Achieving 25% market penetration with a 100-h LDES at 30% RTE would require its power cost to be even lower: approximately 40% of that of a LiB with the same power rating. Similarly, for 8- and 12-h LDES with RTEs below 40%, the power cost targets must drop below the cost of a LiB to capture even 1% of the energy storage market. The energy cost targets have a similar dependence on RTE. Achieving a cost that is 20% of the energy cost of a LiB enables broader adoption of an 8- and 12-h LDES (even at RTEs as low as 30%). However, a 30% efficient 100-h LDES will need to reach about 4% of the energy cost of a lithium battery.

As shown in Figure 2, at lower RTEs, achieving higher market shares (e.g., 25%) requires a substantially greater relative reduction in cost. However, the cost ratios required for different market shares converge as RTE increases. Notably, at 85% RTE, even a modest reduction in LDES cost enables a significant increase in LDES market share from 1% to 25%, particularly for 8- and 12-h LDES energy reservoir sizes, as illustrated in the graphs. Interestingly, for 8-h LDES with 85% RTE, the power and energy cost ratios converge to approximately 2 and 1, respectively. This implies that two LiBs hold roughly the same value as one 8-h LDES with equivalent RTE and power ratings. However, this equivalence does not hold for bigger energy reservoirs, as shown in Figure 2. Specifically, for 12- and 100-h LDES, three LiBs are not equivalent to a single 12-h LDES with the same RTE and power rating, nor are twenty-five 4-h LiBs equivalent to one 100-h LDES. This divergence underscores the unique cost dynamics and system value associated with LDES with larger energy reservoir sizes, which cannot simply be scaled linearly from technologies with smaller energy reservoir sizes.

### Effects of the modeling target year and emissions constraints

**Figure 3:**
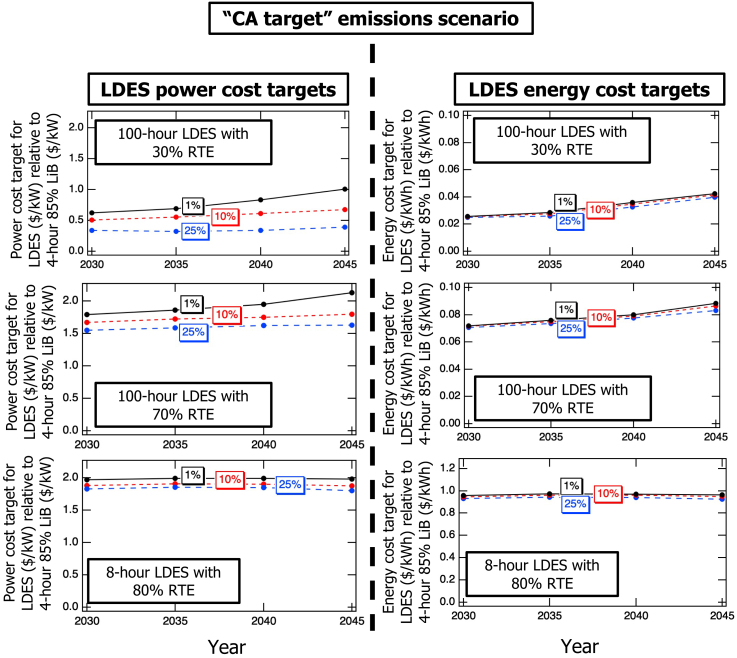
Evolution of LDES cost targets to reach 1%, 10%, or 25% penetration when considering power (left) or energy (right) ratings Calculations assumed the “CA target” carbon emissions scenario for 100-h LDES energy reservoir size for 30% (top) and 70% (middle) RTEs, and for 8-h LDES energy reservoir size for 80% RTE (bottom).

Figure 3 illustrates how the cost targets for power and energy were modeled to evolve into the future for multiple levels of LDES adoption. The analysis considered the three LDES power and energy penetration levels of 1%, 10%, and 25% across the key modeling years of 2030, 2035, 2040, and 2045 under the “CA target” carbon emissions scenario. The graphs show results for 100-h LDES at two RTE levels of 30% and 70% and for 8-h LDES at 80% RTE. The results indicate a slight increase in target power and energy cost ratios for 100-h LDES, regardless of RTE, particularly at low penetration levels, as we move toward future years. For example, by 2045, the power cost of a 100-h LDES with 30% RTE needed to reach 1% market share converges to the cost of a LiB with the same power rating. Meanwhile, for a 100-h LDES with 70% RTE, the power cost target surpasses that of two LiBs with the same power rating, reflecting its higher value to the market. In contrast, for 8-h, 80% LDES, the target year has little to no impact on its cost ratio targets. The cost targets calculated for the energy costs follow a similar trend, with the 100-h storage resources found to have higher cost targets (easier to enter the market) in 2045 relative to 2030, while the cost targets for 8-h, 80% LDES to enter the market change very little relative to LiBs. Overall, these results suggest that LDES with larger energy reservoir sizes becomes more valuable in maintaining reliability as renewables’ penetration grows.

**Figure 4:**
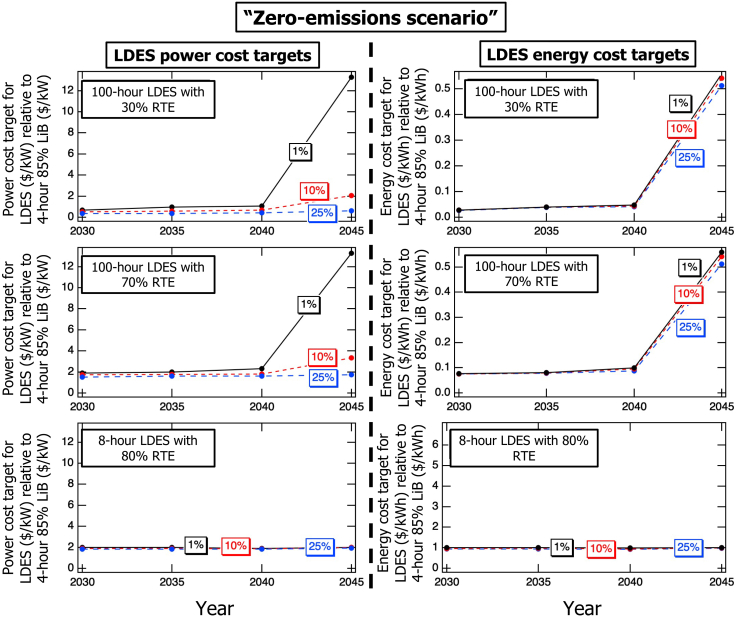
Evolution of LDES cost targets to reach 1%, 10%, or 25% penetration when considering power (left) or energy (right) ratings Calculations assumed the “zero-emissions scenario” for 100-h LDES energy reservoir size for 30% (top) and 70% (middle) RTEs and for 8-h LDES energy reservoir size for 80% RTE (bottom).

Figure 4 presents the same analysis as Figure 3, but under the “zero-emissions scenario.” In this case, the value of 100-h LDES increases by about a factor of ten for 1% market penetration in 2045, even at 30% RTE. A battery-powered energy system must have sufficient power capacity to meet peak demand and an energy reservoir large enough to sustain energy supply during extended periods of low renewable generation. However, relying solely on LiBs for such prolonged periods may lead to power overcapacity, as LiBs' energy capacity cannot be scaled independently of their power capacity. In scenarios that allow even a small amount of carbon emissions, grid resiliency is typically provided by carbon-emitting technologies. However, in the “zero-emissions scenario,” carbon-emitting resources are not allowed to be selected by the model, substantially increasing the value of LDES with an energy reservoir size of 100 h, which, at 1% market share, is chosen to fulfill resiliency requirements rather than for daily use. Referring to example LiB’s costs that are valid for the purposes of this study in the supplemental materials, this corresponds to LDES power cost-ratio targets exceeding 5,000 ($/kW) for small market penetration levels, assuming a cost-recovery period of 10–15 years and a 5% discount rate. These target ratios are comparable to reported power costs of several existing LDES technologies, which span a wide range, from approximately 1,000 ($/kW) to over 10,000 ($/kW), depending on technology and maturity.^14^ This comparison suggests that, under strict zero-carbon constraints and for resilience-driven applications, even current LDES technologies with their high power costs could become economically competitive at limited penetration levels. Similar to the “CA target” carbon emissions scenario, the modeling year has little impact on the value of 8-h LDES in the “zero-emissions scenario.”

**Figure 5:**
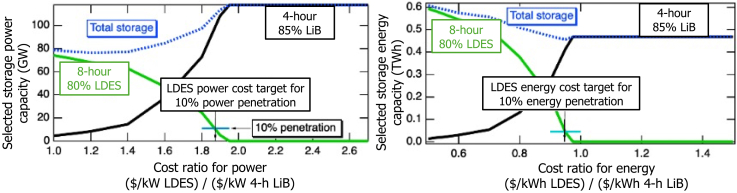
Selection of 8-h 80%-efficient LDES relative to 4-h 85%-efficient LiB as a function of relative cost, considering power (left) and energy (right) ratings The ratio of the green curve to the blue curve identifies the market penetration for the LDES, with 10% penetration marked as an example. The downward arrow to the x axis then derives the LDES cost target for 10% penetration. These examples used the “CA target” carbon emissions scenario for 2045.

As discussed in the STAR Methods Section, our modeling approach explicitly enforces hourly energy balance without imposing planning-reserve-margin (PRM) constraints or assigning electric-load-carrying-capacity (ELCC) values. Therefore, as mentioned, the notable increase in the value of a small penetration (1%) of 100-h LDES in 2045 under the “zero-emissions scenario” is attributed to resilience, which is the ability to reliably meet projected demand during extended high-load periods and/or prolonged renewable-electricity generation shortfalls. In alternative energy systems or modeling methods where resiliency receives greater emphasis, such as those incorporating PRM constraints and assigning ELCC values to the resources, the value of 100-h LDES, especially at small penetration levels, is likely to be even higher.

### Significance and applicability of the results

The results of this example implementation of our model using California’s solar-dominated grid show how the model can elucidate the evolving role of energy storage in future electric grids, from the perspectives of both cost efficiency and system reliability and resiliency. From the cost-efficiency standpoint, the modeling suggests that increasing the energy reservoir size up to 8 h increases the value of the storage proportionally to the size of the energy reservoir. Increasing the reservoir size of LDES beyond 8 h provides limited additional value from the cost-efficiency standpoint when executed in a model with perfect foresight of the weather and load profiles. From the standpoint of system reliability and resiliency, the value of 100-h LDES for a small market fraction may increase by a factor of 5 or even 10, as seen for the “zero-emissions scenario,” where no carbon-emitting backup generation is allowed. LDES with 100-h reservoir sizes may also increase the resilience of microgrids designed to provide back-up power when grid electricity is unavailable. Quantifying that value was outside the scope of this study.

These findings from the analyzed example case broadly apply to many highly populated regions, particularly those in sunbelt areas near the equator, where solar resources are abundant.^20^ In such grids, storage systems with smaller energy reservoir sizes of up to 8 h, as shown in this study, are highly effective for addressing diurnal supply-demand imbalances. Additionally, limited storage deployment with larger reservoir sizes (e.g., 100 h, as shown in this study) can enhance grid resiliency, especially under the “zero-emissions scenario,” as discussed earlier. However, these configurations may differ for grids with distinct characteristics. For instance, Mahmud and Kurtz^21^ showed that different variability and intermittency patterns in wind-dominated grids could drive the adoption of 100-h storage over 8-h storage. Moreover, Staadecker et al.^4^ systematically quantified the value of LDES across 39 grid scenarios in the Western Interconnection and showed that LDES value is highly system-dependent; for instance, winter-dominant and winter peaking regions favor LDES with larger reservoir sizes (10–20 h and beyond), while solar-dominant grids are best supported by 6–10 h storage (consistent with this study’s finding); furthermore, they found that for grids with low hydropower availability and limited transmission expansion, the LDES value could significantly increase. Although we didn’t apply our proposed method to wind-dominant or transmission-limited grids, our proposed modeling approach can easily be applied to such situations.

### Takeaways and case study insights

This study proposed a method to quantify the competitiveness of LDES relative to 4-h LiB, the primary competitor in grid-scale storage. In this study, LDES is defined as a storage system in which the energy reservoir can be scaled independently of the power conversion hardware. Accordingly, we introduced the term “energy reservoir size (hours)” rather than the traditional “duration” terminology for LDES. Then, the proposed cost target method was applied to an example case of California’s solar-dominated, renewable-energy-driven grid using a full-resolution, 8,760-hour-per-year linear capacity optimization model, and the results demonstrated that:

• The cost-target approach requires additional computational effort but guides a technology developer to identify the price relative to LiB needed for a specific type of LDES to capture market share.
• For the example California’s solar-dominant grid, LDES with 8-h energy reservoir size represents a critical balance point, balancing both power and energy cost competitiveness, demonstrating the method’s ability to answer the question of “as I am developing an LDES product, what energy reservoir size is likely to be most marketable?” As penetration increases, this optimal point shifts slightly toward LDES with smaller energy reservoir sizes (for instance, 7-h LDES at higher LDES market shares).
• The power value of LDES with high RTEs such as 70% and 80% increases approximately linearly with the size of the energy reservoir up to about 8 h. Beyond this point, additional increases in the energy reservoir size result in a slower growth in the LDES value. More specifically, the cost competitiveness of one 8-h LDES is approximately equivalent to two 4-h LiBs with the same power capacity and RTE. This cost competitiveness remains consistent across different modeling target years (e.g., 2045 vs. 2030) and penetration levels (e.g., 25% vs. 1%). However, this relationship does not extend to larger energy reservoir LDES; for instance, the cost competitiveness of one 12-h LDES unit is not equivalent to three 4-h LiBs with the same power rating and RTE. Again, these results demonstrate the value of our method in guiding product design and development.
• In 2045, 100-h LDES is projected to enter the market at a price similar to the price required to enter the market today in the absence of strict carbon-emissions constraints. However, under a zero-carbon emissions scenario, a small penetration of 100-h LDES, even with low efficiencies (e.g., 30%), can be selected by the model at a ten times higher price, making it easier to enter the market. This potentially could enable the deployment of even existing LDES technologies despite their currently higher power-related costs, as discussed in this study. This is because, in zero-carbon grids, 100-h LDES is primarily deployed for grid resiliency, a role typically filled by carbon-emitting resources in scenarios that allow any emissions. Moreover, this value increase is likely to be even greater in grid or modeling methods that explicitly emphasize resiliency, such as those incorporating PRM constraints, unlike the hourly energy balance approach without PRM assumptions employed in this study.

Our example study of California’s solar-dominated grid and extension of our study to other grid configurations can provide practical insights for LDES developers, helping them determine whether increasing the LDES energy reservoir size can enhance the value of the LDES under specific performance and grid constraints. In other words, the findings can guide developers in optimizing the energy reservoir size, which directly influences the energy capacity cost of their product. For regulators and utilities, the power- and energy-cost ratio target results can inform procurement planning and capacity market or incentive design, by identifying the cost ranges at which LDES becomes competitive and begins to deliver system-level benefits such as reliability improvement and emissions reductions.

### Limitations of the study

As noted, the case study presented here minimizes total system cost under assumptions of perfect foresight and fully deterministic inputs, such as for load and other renewable generation, choices made intentionally to maintain computational efficiency and conceptual clarity. Using LDES to address grid outages or unanticipated weather using stochastic modeling is expected to exhibit different results and could lead to a higher value for LDES with larger energy reservoir sizes. Furthermore, as noted above, the case study presented here reflects a predominantly solar-dominated and summer-peaking system, while the winter-peaking and wind-dominated systems tend to favor the deployment of LDES with larger reservoir sizes. Finally, the analysis does not explicitly model extreme weather events or resilience-driven outages, which could further increase the value of LDES. The methodology proposed in this study, which is shown in Figure 5 and demonstrated in the STAR Methods section, could be adapted to analyze all these grids with different characteristics.

## Resource availability

### Lead contact

Further information and requests for resources and input files should be directed to and will be fulfilled by the lead contact, Farzan ZareAfifi (fzareafifi@ucmerced.edu).

### Materials availability

This study did not generate any new unique reagents.

### Data and code availability

• This paper uses publicly available model assumptions data, which are all listed in the key resources table.
• This paper uses the publicly available RESOLVE capacity optimization model, and the access link to the RESOLVE package is provided in the key resources table.
• Any additional information required to reanalyze the data reported in this paper or the output data from the model is available from the lead contact upon request.

## Acknowledgments

The authors would like to thank R. Go for guidance on the RESOLVE software, MY Abido for his help with the early RESOLVE calculations, and J. Reagan, T. Le, and P.A. Sánchez-Pérez for their help in code development and data gathering. This work was partly supported by the 10.13039/100004805California Energy Commission [EPC-19-060]. This document was prepared as a result of work sponsored by the 10.13039/100004805California Energy Commission. It does not necessarily represent the views of the Energy Commission, its employees, or the State of California. The Energy Commission, the State of California, its employees, contractors, and subcontractors make no warranty, express or implied, and assume no legal liability for the information in this document; nor does any party represent that the use of this information will not infringe upon privately owned rights. This report has not been approved or disapproved by the Energy Commission nor has the Energy Commission passed upon the accuracy of the information in this report.

## Author contributions

F.Z.: formal analysis, visualization, writing, review and editing, investigation, and validation. Z.M.: software, formal analysis, data curation, review and editing, and validation. S.K.: conceptualization, methodology, software, formal analysis, visualization, writing, data curation, review and editing, investigation, validation, project administration, supervision, and fund acquisition.

## Declaration of interests

The authors declare no competing interests.

## STAR★Methods

### Key resources table

**Table:** 

REAGENT or RESOURCE	SOURCE	IDENTIFIER
**Deposited data**		

2019 RESOLVE Package User Manual	California Public Utilities Commission	https://www.cpuc.ca.gov/-/media/cpuc-website/divisions/energy-division/documents/integrated-resource-plan-and-long-term-procurement-plan-irp-ltpp/2019-2020-irp-events-and-materials/resolve-user-guide---public-release-20191106.pdf
Draft 2023 inputs and assumptions document for the integrated resource planning (IRP) process	California Public Utilities Commission	https://www.cpuc.ca.gov/-/media/cpuc-website/divisions/energy-division/documents/integrated-resource-plan-and-long-term-procurement-plan-irp-ltpp/2023-irp-cycle-events-and-materials/draft_2023_i_and_a.pdf
Fact sheet: Proposed decision adopting 2023 preferred system plan	California Public Utilities Commission	https://www.cpuc.ca.gov/-/media/cpuc-website/divisions/energy-division/documents/integrated-resource-plan-and-long-term-procurement-plan-irp-ltpp/2022-2023psp_pd_2pager_ver2.pdf

**Software and algorithms**		

2021 RESOLVE Package	California Public Utilities Commission	https://files.cpuc.ca.gov/energy/modeling/2021%20PSP%20RESOLVE%20Package.zip

### Method details

In this section, we describe the modeling method and key assumptions used in this study, and outline the approach adopted to quantify the cost thresholds that LDES technologies, relative to 4-h LiB, must achieve to capture specific shares of the energy storage market. Additionally, this section includes sensitivity analysis conducted to evaluate and justify the simplifying assumptions underlying our primary modeling approach for applying the model to the example application of California’s solar-dominated and renewable-energy-driven grid.

To develop our approach of modeling the adoption of different types of storage, we used the publicly available RESOLVE capacity optimization model,^22^ a Python-based linear optimization tool designed to co-optimize resource buildout and hourly dispatch within California’s renewable-energy-driven grid,^23^ which is expected to be solar-dominated as shown by previous studies; for example, Jacobson et al.^24^ projected that solar PV generation could supply approximately 40% of California’s annual electricity demand in 2050. RESOLVE is publicly available (to download, see^22^), and running a single-target-year, hourly resolution simulation (8,760 timesteps), such as the calculations that we conducted for the case study in this analysis, typically required 30–120 min on a standard workstation equipped with a modern multicore CPU and using the Gurobi solver. Runtime depended on many parameters such as model size, zonal structure, reserve constraints, and solver configuration. RESOLVE is spatially resolved at the zonal level and not at the nodal or substation level. The version that we used in this study represented California and the surrounding Western Interconnection system using seven zones.^22^ RESOLVE minimizes the total system cost, accounting for capital expenditures, fixed and variable operation and maintenance (O&M) costs, start-up and shutdown costs, fuel costs, and hurdle costs for transmission. The cost function is structured as in Equation 1; the expanded formulation is provided in the supplemental materials.

(Equation 1) $$Cost\;Function=\sum_{r}\left(C_{r}^{fix}+C_{r}^{var}+C_{r}^{start/\;shutdown}+C_{r}^{fuel}\right)+\sum_{s}C_{s}^{curtailment}+\sum_{t}C_{t}^{transmission}+\sum_{p}C_{p}^{penalty}$$

where.

• r indexes power generation and storage resources.
• $C_{r}^{fix}$ represents the total fixed costs, including capital costs and fixed O&M.
• $C_{r}^{var}$ represents variable O&M costs, summed for all hours of the year.
• $C_{r}^{start/\;shutdown}$ represents the start-up and shutdown costs.
• $C_{r}^{fuel}$ represents the cost of fuel procurement.
• s indexes variable renewable resources.
• $C_{s}^{curtailment}$ represents the cost of curtailing excess generation. RESOLVE applies curtailment costs only to existing resources whose power-purchase agreements obligate payment even when energy is curtailed. Newly built resources are modeled without curtailment penalties, and curtailment does not represent a physical cost but rather a contractual payment obligation associated with certain existing assets.
• t indexes transmission assets.
• $C_{t}^{transmission}$ represents the hurdle costs for transmission expansion or use.
• p indexes penalty terms.
• $C_{p}^{penalty}$ represents penalties for unserved energy, overgeneration, and unserved reserves.

Model inputs included resource capacity and transmission constraints, solar and wind resources’ hourly generation profiles, hourly load profiles for multiple zones as described in the supplemental materials, fuel and resource costs, policy requirements, and other general assumptions of RESOLVE.^23^ To develop the generation profiles, we used actual weather data from 2007. To assess the effect of weather variability on the results, we also performed calculations using weather data from 2008 to 2009, discussed in the supplemental materials. The years 2007, 2008, and 2009 include hourly load-profile datasets developed by the State of California and used by RESOLVE for capacity optimization modeling purposes, as mentioned in the inputs and assumptions of the State of California’s 2022–2023 Integrated Resource Plan (IRP) report.^25^

For solar generation, a single-axis tracking system with no tilt angle was assumed for all profiles as most new projects in the U.S. have recently chosen single-axis tracking over fixed-tilt racking,^26^ although alternative solar mounting configurations could impact capacity optimization decisions.^27^^,^^28^ For all generation resources other than wind, solar, and hydropower, RESOLVE optimizes dispatch decisions while considering ramping and fuel availability constraints; this includes bioenergy/biogas technologies, which were incorporated into the RESOLVE resource library. However, RESOLVE did not select additional bioenergy/biogas capacity in any of the modeled scenarios in the discussed case study, as these technologies were not cost-competitive compared with other available clean resources.^29^ For hydropower, we used historical generation profiles obtained by comparing the hydropower generation for each year from 2019 through 2021 and selecting the lowest generation year for each region. Ideally, hydropower can be used as a dispatchable generator to help balance electricity supply and demand. We chose the historical fixed dispatch profiles to provide practical, but low, generation profiles for what we anticipate to be a worst-case year.^30^^,^^31^ This ensures that the dispatch profile remains unchanged and cannot be adjusted by the model, thus clarifying competition between LDES and LiB without confounding effects from changing the hydropower generation profile. Additionally, we verified that this approach did not meaningfully break weather interconnectivity for our California’s renewable-energy-driven grid case study: of the 48 MW of operating hydropower capacity represented in our RESOLVE model, about 45 MW are located in regions for which 2021 was identified as the driest year. Finally, negative-emissions technologies such as direct air capture and bioenergy with carbon capture were not modeled in this study. Incorporating such technologies would require substantial expansions to the system boundary and was therefore considered outside the scope of this analysis.

In its standard preferred portfolio configuration,^22^ RESOLVE incorporates PRM constraints along with effective ELCC values assigned to individual resources. However, we found that the standard ELCC values for resources selected in our 2045 modeling scenario were not well-suited for our analysis. Thus, we did not include the PRM constraint (which uses the ELCC values) and, instead, directly assessed system reliability by requiring adequate power for every hour of the year. Consequently, neither explicit PRM constraints nor resource-specific ELCC values were applied. Instead, each resource’s capacity contribution, including LDES, was inherently evaluated based on its actual dispatch and ability to shift energy to meet load across all hours. To maintain operational reliability, we required spinning reserve of 3% of hourly gross load, along with an additional 5% of gross load reserved for upward and downward regulation, upward and downward load-following reserves, and frequency response (1% each) within the California Independent System Operator (CAISO) zone for every hour of the modeled year. Consequently, our focus in developing our approach and analyzing the California’s solar-dominated grid as a show-case application of the method is primarily on identifying cost-optimal pathways to reliably meet projected demand, rather than addressing scenarios designed for resilience or extreme reliability events. Nevertheless, the approach can be adapted for analyzing applications emphasizing resiliency.

In this study, we analyzed two carbon-emissions constraint scenarios: the “zero-emissions scenario,” which applies a zero carbon emissions constraint for electricity generation in California (CAISO) in 2045, and the 38 million metric tonnes (MMT) carbon emissions scenario by 2030, which estimates a zero-carbon requirement for retail electricity in 2045 while allowing emissions for non-retail electricity generated in California, as detailed in the supplemental materials. The 38 MMT by 2030 scenario has been used by the state of California in official long-term energy planning and capacity optimization studies conducted by the California Energy Commission,^32^^,^^33^ and aligns with executive-level climate policy directives that establish binding greenhouse gas reduction targets for the electricity sector.^34^ This scenario is referred to as the “CA target” carbon emissions scenario in this study.

Regarding RESOLVE’s outputs, the buildout data for the LiB and LDES resources are most relevant to this study and were used extensively in the analysis. For context, across modeled cases, solar and wind constitute approximately 50–60% of total new resource buildouts in 2045, depending on the carbon-emissions scenario, LDES RTE, and energy reservoir size. Most of the remaining new resources consist of storage resources that complement the variable output of solar and wind generation. Importantly, the selection of capacity expansion of solar and wind is relatively independent of the selected mix of LDES and LiB storage, as shown in the supplemental materials. Please refer to the supplemental materials for more information on RESOLVE’s inputs, constraints, assumptions, buildout of non-storage resources, and generation dispatch of all resources for the analyzed case study.

The storage system configuration used in our RESOLVE modeling consisted of a single LDES technology (with design parameters varied across simulations) alongside 4-h LiB in each simulation set. To assess the effect of LiB with different durations, we also performed calculations using 1- and 2-h LiB, discussed in the supplemental materials. As stated in the Introduction, in this study LDES is defined as a storage system where the energy reservoir can be expanded separately from the power conversion equipment, enabling energy delivery for durations of an arbitrary number of hours. Therefore, in this study, instead of using the term “duration (hours),” we referred to it as “LDES energy reservoir size (hours)” to represent the energy delivery duration at the rated power. As outlined in the Introduction, in this study we evaluated the value of LDES in future renewable-energy-driven grids relative to 4-h LiBs. The 4-h duration represents the most widely deployed and historically dominant grid-scale battery configuration and is well suited for providing capacity during summer peak demand periods, as demonstrated in a report by the National Renewable Energy Laboratory (NREL).^10^ We also examined the sensitivity of the results to alternative LiB configurations (1-h and 2-h), discussed in the supplemental materials. In the discussed case study, we evaluated energy reservoir sizes greater than 4 h^35^ in order to assess how LDES could economically compete with LiBs for different applications, especially diurnal energy shifting. The RTE of LiB was assumed to be 85% throughout the analyzed case study, based on the current observed performance in utility-scale storage plants.^36^^,^^37^ The annualized cost of 4-h LiB was considered 66.2 ($/kW-year) by 2045, based on the assumptions outlined in the 2023 IRP report published by the state of California.^25^ To align with this assumption, any combination of capital cost, O&M cost, and cost recovery period that results in the specified annualized cost can be considered valid for the purposes of this analysis. For more information on how the all-in annualized power cost is calculated and also for example combinations of these cost parameters, please see the supplemental materials.

#### Cost target approach

Figure 5 illustrates the storage selection by the model for 4-h LiB and 8-h LDES on the y axis, depicted for power capacity (left) and energy capacity (right) for the 2045 target year. The x axis represents the cost ratio of an 8-h 80%-efficient LDES to a LiB, expressed in ($/kW)/($/kW) (left) and ($/kWh/$/kWh) (right). At high costs of LDES, the model selects only LiBs. However, as the cost for LDES decreases and passes a specific threshold, LDES becomes more competitive and begins capturing market share. At very low costs of LDES, the model exclusively selects LDES, phasing out LiBs entirely. The blue curve in both plots shows the total storage capacity, power on the left, and energy on the right, as the sum of the LiBs (black curve) and LDES (green curve) capacities. The LDES market share discussed in the subsequent sections was calculated as the LDES capacity (green curve) divided by the total storage capacity (blue curve) at each cost target. As an example, the points at which LDES has 10% power and energy penetrations are depicted in the left and right graphs in Figure 5, respectively. We present both power and energy cost target results, as it is important to evaluate both when applying this methodology to different grid configurations. In some cases (e.g., 100-h LDES in our case study), the power and energy cost targets results are different, and interpreting only one would provide an incomplete picture.

This methodology derives the cost target to achieve a specific penetration level. However, accurately determining the cost target for a specific LDES penetration requires multiple runs of the capacity optimization model under varying LDES cost assumptions. Relying on linear interpolation without sufficient model runs risks significant errors, especially in the transition region, where small changes in the LDES cost assumptions affect storage selection by the model remarkably. Therefore, making smart LDES cost assumptions while utilizing the cost target approach for analyzing LDES competitiveness is critical, as it can reduce the computational load significantly. As mentioned, while this approach is adaptable to other scenarios, including those with multiple storage options, this study focuses solely on these two storage types for California’s solar-dominated grid as our demonstration case.

Finally, the demonstrated method in this section is for quantifying cost targets needed for LDES adoption under defined modeling assumptions rather than determining the universally optimal LDES energy reservoir size or defining the full value stack of LDES across all contexts. The methodology can be adapted to a wide range of grid conditions and market environments, such as systems where resilience and multi-day outages are primary drivers of value.

### Quantification and statistical analysis

There are no quantification or statistical analyses to include in this study.
