# Supplementary material for: Path to future markets: Method for quantifying long-duration energy storage competitiveness
Source: iScience. 2026 Apr 15;29(6):115751. doi: 10.1016/j.isci.2026.115751 (PMC13186050; doi:10.1016/j.isci.2026.115751)
Supplement: Document S1. Figures S1–S7, Tables S1–S8, and Methods S1–S4 [file mmc1.pdf]

iScience, Volume *29*

Supplemental information

Path to future markets: Method for quantifying long-duration energy storage competitiveness

Farzan ZareAfifi, Zabir Mahmud, and Sarah Kurtz

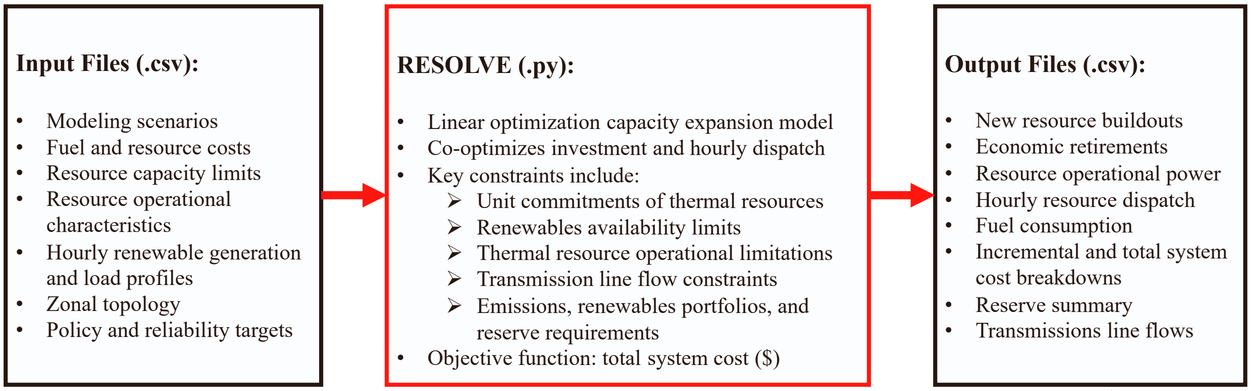

Fig S1. High-level summary of RESOLVE model's inputs, constraints, and outputs[1]

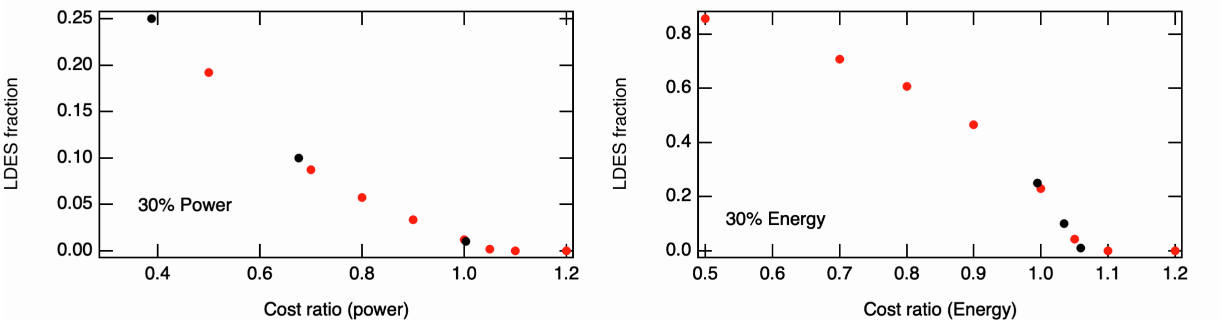

Fig S2. LDES fraction of total storage selected by model as a function of relative cost considering power (left) and energy (right) ratings. The red data points show the output of the RESOLVE optimization. The black points were interpolated (or extrapolated) from the red data points. The black points for the 1%, 10%, and 25% market share were then plotted on other graphs as a function of the efficiency and other parameters. The calculations are for 100-h, 30%-efficient LDES.

[1] https://www.cpuc.ca.gov/-/media/cpuc-website/divisions/energy-division/documents/integrated-resource-plan-and-long-term-procurement-plan-irp-ltpp/2019-2020-irp-events-and-materials/resolve-user-guide---public-release-20191106.pdf

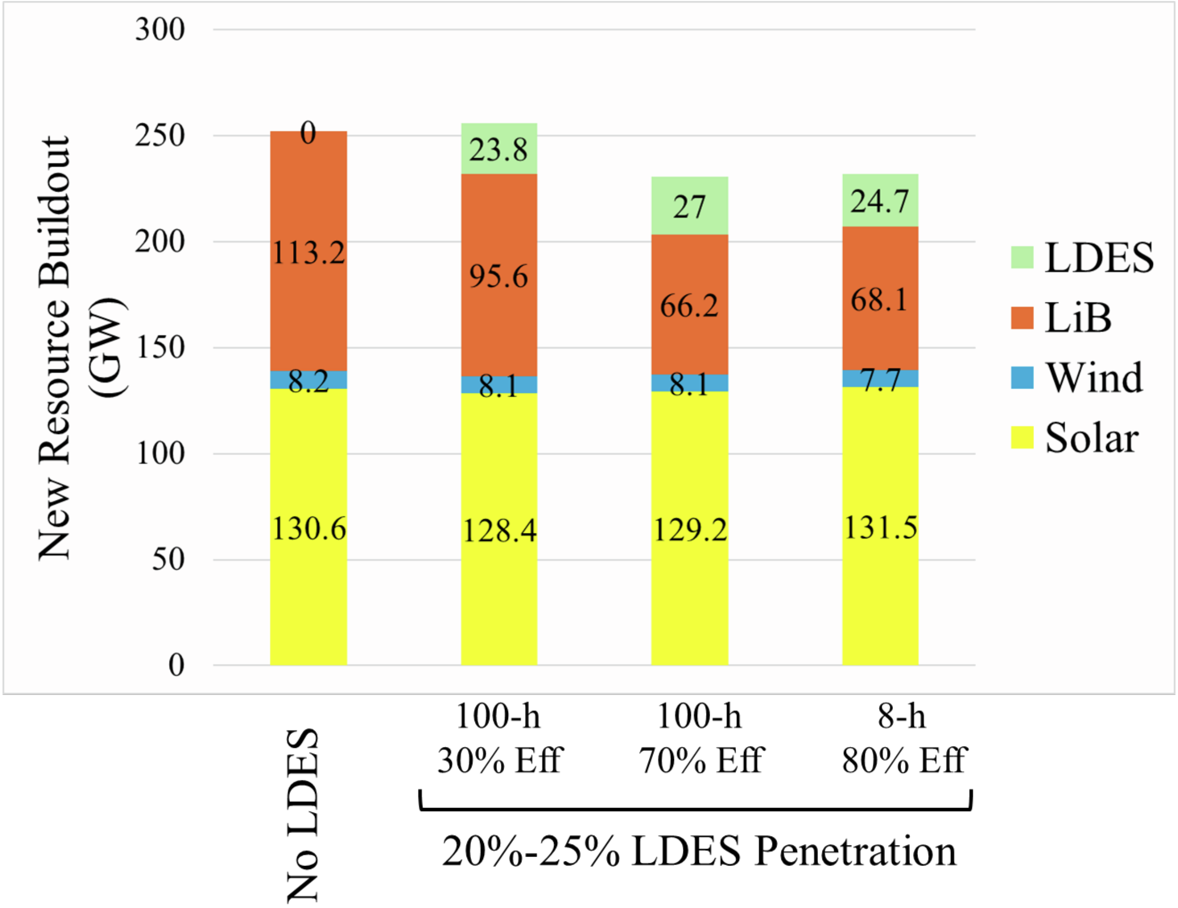

Fig S3: New resource buildout (GW) for 0% and 25% penetration levels (in the total storage buildout) of 100-hour LDES (30% and 70% RTEs) and 8-hour LDES (80% RTE) in 2045. RESOLVE optimizes resource selection based on cost and the defined power potential of each resource. The graph indicates that the selection of wind and solar capacity remains approximately identical across these four cases. However, as LDES replaces LiB, the total power buildout varies depending on the LDES RTE. Additionally, small, consistent amounts of offshore wind and geothermal (<0.2 GW) are selected across all four cases but are not shown in the figure for clarity.

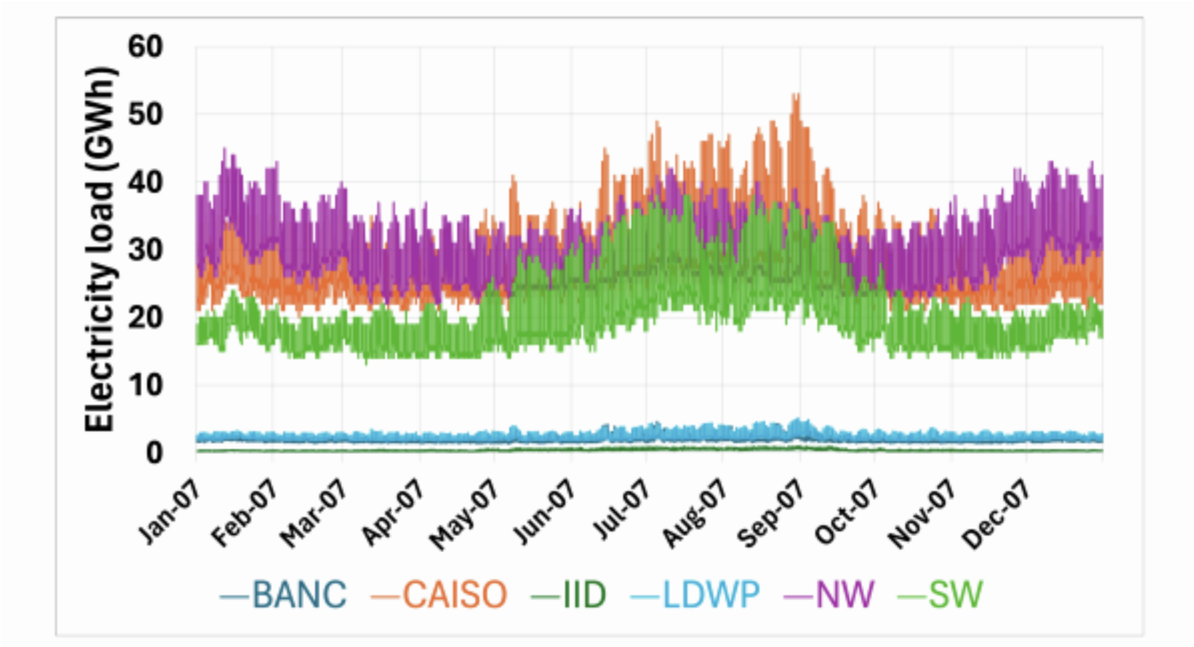

Fig S4: Hourly total 2045 load profiles for the six modeled zones using 2007 input data in RESOLVE simulations

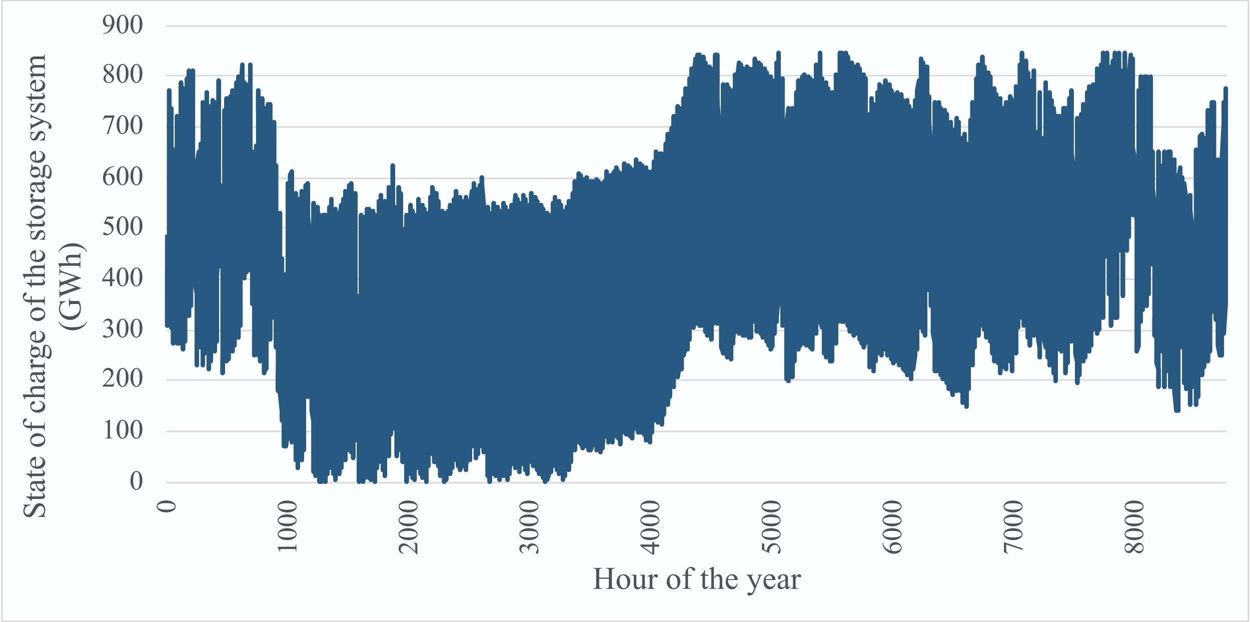

Fig S5: State of charge of all modeled storage resources in 2045 in all of the modeled zones

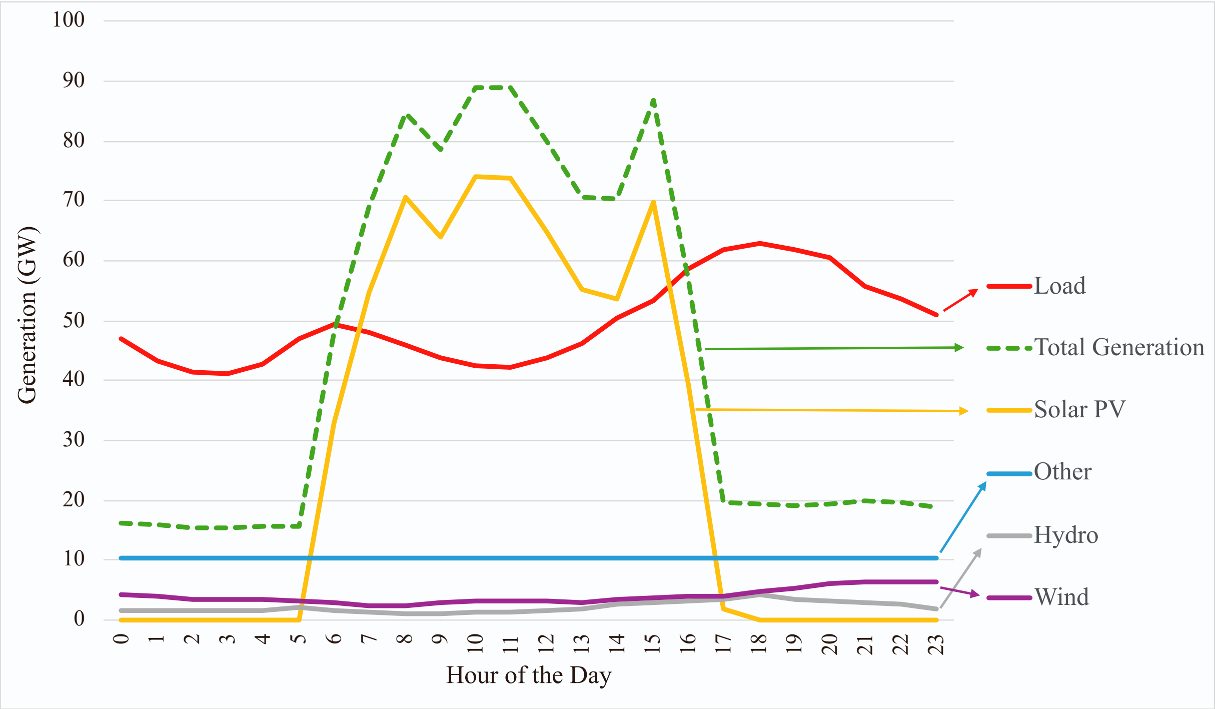

Fig S6: Generation hourly profiles in Sep 21st, 2045 (Autumnal Equinox) for resources in CAISO; "other" resources in this figure include geothermal, natural gas, coal, nuclear, and biomass

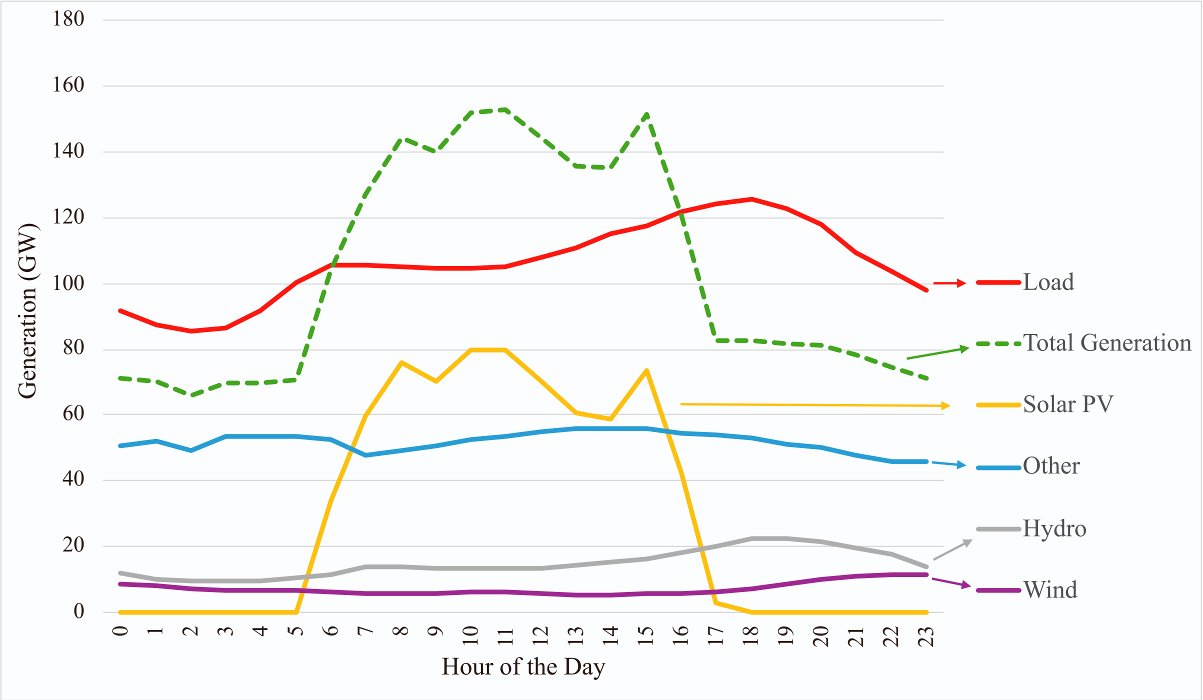

Fig S7: Generation hourly profiles in Sep 21st, 2045 (Autumnal Equinox) for resources in all of the modeled zones; "other" resources in this figure include geothermal, natural gas, coal, nuclear, and biomass

Table S1: Total potential of each resource in California used in RESOLVE and the new power buildouts in Fig. S3. As indicated, the new buildout remains consistently below the defined resource potential. The model does not select the buildout of biomass resources. Additionally, hydropower and pumped hydro were not offered as buildout options in the model.

Resource	Potential (GW)	New Buildout in Fig. S3 (GW)			
		No LDES	100-h 30% RTE LDES	100-h 70% RTE LDES	8-h 80% RTE LDES
Solar	579.7	130.6	128.4	129.2	131.5
Onshore wind	38.1	8.2	8.1	8.1	7.7
Offshore wind	4.7	0.2	0.2	0.2	0.2
Geothermal	2.3	0.1	0.1	0.1	0.1
Biomass	0	0	0	0	0

Table S2: All-in annualized cost ranges ($/kW-yr) for different resources used in RESOLVE for 2045. The costs for earlier years were similar, but a little lower for each. Any combination of capital cost, O&M cost, and cost recovery period that results in the specified annualized cost may be implied by these annualized costs (Examples provided in the Table).

**Resource**	**Examples of Valid Cost Combinations**			**All-in Annualized Cost ($/kW-yr)**
	**Capital Cost ($/kW)**	**O&M Cost ($/kW-yr)**	**Cost Recovery Period (yr)**	
Solar	570	10	20	Min: 56<br>Max: 58
	710	10	30	
	600	10	20	
	740	10	30	
Onshore wind	1080	36	20	Min: 123<br>Max: 224
	1340	36	30	
	2190	36	20	
	2700	36	30	
	2340	36	20	
	2890	36	30	
Geothermal	4075	135	20	Min: 462<br>Max: 554
	5025	135	30	
	5220	135	20	
	6440	135	30	
Offshore Wind	2180	44	20	Min: 219<br>Max: 224
	2690	44	30	
	2240	44	20	
	2770	44	30	
4-h Lithium Battery	450	8.3	10	66.2
	600	8.3	15	

Table S3: CAISO greenhouse gas (GHG) emission constraints in million metric tons (MMT) used in our modeling

Scenario	2030	2035	2040	2045
Zero-emissions	27	18	9	0
CA target	31.1	24.8	18.5	12.3

Table S4: Annual load forecast (TWh/year) for the modeled zones in 2045

Zone	Annual energy forecast (TWh/year)
BANC	18.4
CAISO	400.8
IID	3.8
LDWP	22.6
NW	273.7
SW	183.5
Total	902.8

Method S1: Lithium-ion batteries all-in annualized cost calculation and examples

Capital cost = C_{cap} (\$/kW)
Fixed O&M cost = C_{FOM} (\$/kW-yr)
Discount rate = r (per year; 5% in this study)
Cost recovery period = N (yr)
Capacity recovery factor (CRF):

$$\mathrm{CRF} = \frac{r \times (1+r)^N}{(1+r)^N - 1}$$

The all-in annualized power cost becomes:

$$C_{power,\ annual,\ all\text{-}in} = C_{cap} \times \mathrm{CRF} + C_{FOM}$$

$$C_{energy,\ annual,\ all\text{-}in} = \frac{\mathrm{Cpower{,}annual{,}all{-}in}}{Storage\ duration}$$

Table S5. Example cost components for 4-hour LiBs with 85% RTE valid in the study: two example scenarios resulting in the same assumed annualized cost used in this study. The discount rate is assumed to be 5%. Note that the all-in annualized energy cost (in \$/kWh-yr) is ¼ of the all-in annualized power cost (in \$/kW-yr), reflecting the difference when the battery cost is reported as calculated for the energy rating or for the power rating.

	Capital cost (\$/kW)	O&M cost (\$/kW-yr)	Cost recovery period (yr)	All-in annualized power cost (\$/kW-yr)	All-in annualized energy cost (\$/kWh-yr)
Scenario 1	450	8.3	10	66.2	16.6
Scenario 2	600	8.3	15		

Method S2: Effect of weather year data on cost targets

As mentioned in the STAR Methods Section, we used actual weather data from 2007 to develop the generation profiles for the analyses presented in the case study Section. To evaluate the sensitivity of our findings to interannual weather variability, we also performed simulations using weather data from 2008 and 2009. Table S6 provides a side-by-side comparison of the modeled energy cost targets required for 8-hour LDES with 80% RTE to achieve energy market penetration levels of 1%, 10%, and 25%, relative to 4-hour LiB with 85% RTE, across these three weather years. As indicated by Table S6 and based on other cases that we assessed, differences across these weather years are typically within about 1%. Therefore, to reduce computational complexity and streamline visualization, the results presented in other parts of this study are based exclusively on modeling conducted with the 2007 weather dataset.

Table S6. Energy cost targets in ($/kWh)/($/kWh) calculated for 8-hour LDES with 80% RTE relative to 4-hour LiB with 85% RTE using the three weather years. The reported ± values reflect the interpolation uncertainty arising from estimating the LDES energy cost target at the exact penetration level from discrete model outputs.

LDES energy reservoir size	LDES energy penetration	Weather year		
		2007	2008	2009
8-hour	1%	0.97 ± 0.01	0.98 ± 0.02	0.97 ± 0.02
	10%	0.948 ± 0.005	0.945 ± 0.01	0.945 ± 0.02
	25%	0.926 ± 0.01	0.92 ± 0.02	0.92 ± 0.02

Method S3: Effect of lithium-ion battery duration on cost targets

As mentioned, the primary analysis in this study evaluated the cost targets of LDES relative to 4-hour LiB. To assess the sensitivity of our findings to the choice of LiB duration, we also conducted simulations using 1-hour and 2-hour LiB durations. For consistency in this sensitivity analysis, the all-in annualized energy cost was held constant at 16.6 ($/kWh-yr) across the three durations. Consequently, the resulting assumed all-in annualized power cost for 1-, 2-, and 4-hour LiB was 16.6, 33.1, and 66.2 ($/kW-yr), respectively. Table S7 compares the modeled energy cost targets required for 8-hour LDES with 80% RTE to achieve energy market penetration levels of 1%, 10%, and 25%, relative to LiB of 1-, 2-, and 4-hour durations. As shown in Table S7 and confirmed by additional cases we assessed, differences among these cases were found to be negligible and fall within the uncertainty values. Therefore, to simplify computations and visualization, the analyses presented throughout the main results of this study rely exclusively on the 4-hour LiB duration assumption.

Table S7. Energy cost targets (in \$/kWh)/(\$/kWh) calculated for 8-hour LDES with 80% RTE relative to LiB with 1-, 2-, and 4-hour duration and 85% RTE; The reported ± values reflect the interpolation uncertainty arising from estimating the LDES energy cost target at the exact penetration level from discrete model outputs.

LDES energy reservoir size	LDES energy penetration	LiB duration with 85% RTE		
		1-hour	2-hour	4-hour
8-hour	1%	0.98 ± 0.02	0.98 ± 0.02	0.97 ± 0.01
	10%	0.945 ± 0.005	0.945 ± 0.005	0.948 ± 0.005
	25%	0.926 ± 0.02	0.926 ± 0.02	0.926 ± 0.01

Method S4: Objective Function for Optimizations

In the main paper, a brief description of the objection function was specified. Here we expand on this description, adding equations for each term in Eq. 1.

$$Cost\ Function = \sum_r (C_r^{fix} + C_r^{var} + C_r^{start/\ shutdown} + C_r^{fuel}) + \sum_s C_s^{curtailment} + \sum_t C_t^{transmission} + \sum_p C_p^{penalty} \qquad (1)$$

where:

- r indexes power generation and storage resources.
- C_r^{fix} represents the total fixed costs, including capital costs and fixed O&M. (see Fig. S3), (Eq. S1)
- C_r^{var} represents variable O&M costs, summed for all hours of the year. (Eq. S2)
- $C_r^{start/\ shutdown}$ represents the start-up and shutdown costs. (Eq. S3)
- C_r^{fuel} represents the cost of fuel procurement. (Eq. S4)
- s indexes variable renewable resources.
- $C_s^{curtailment}$ represents the cost of curtailing excess generation. RESOLVE applies curtailment costs only to existing resources whose power-purchase agreements obligate payment even when energy is curtailed. Newly built resources are modeled without curtailment penalties, and curtailment does not represent a physical cost but rather a contractual payment obligation associated with certain existing assets. (Eq. S5)
- t indexes transmission assets.
- $C_t^{transmission}$ represents the hurdle costs for transmission expansion or use. (Eq. S6)
- p indexes penalty terms.
- $C_p^{penalty}$ represents penalties for unserved energy, overgeneration, and unserved reserves. (Eq. S7)

The cost of building and operating generators and storage was calculated according to

$$Asset\ costs = \sum_r C_r^{fix} = \sum_i \sum_r \left[C_r \left(\frac{\$}{MW}\right) + C_r^{FOM} \left(\frac{\$}{MW}\right)\right] \times \boldsymbol{Cap_r}(MW) \qquad (S1)$$

Where i indicates the year and r indicates the selected resource (either generator or storage). C_r is the annualized procurement cost as well as fixed operating cost of asset r and Cap_r is the capacity of asset r that was selected to be built. The annualized asset cost depended on the year of installation, but that cost was then extended into the future in alignment with how a standard mortgage would be implemented. Thus, an asset installed in 2045 was modeled to add a cost that was based on the anticipated selling price in 2045, calculated as the annualized cost with cost recovery factor as described above. Then that annualized cost was paid each year for 20 years into the future. Note that assets that provide storage could have their costs calculated based on the MWh of storage built instead of on the MW built, but each candidate storage asset had a predefined duration so there was no need to calculate the cost based on both the MW and the MWh installed. For simplicity, we have described the calculation for all assets based on their MW capacity, in alignment with how the data are presented in Fig. S3.

The variable operating costs were calculated according to

$$Variable\ operating\ costs = \sum_r C_r^{var} = \sum_i \sum_r \left[\sum_{h=1}^{8760} C_r^{VOM}\left(\frac{\$}{MW}\right) \times \boldsymbol{Cap_r^h}(\boldsymbol{MW})\right] \qquad \text{(S2)}$$

Where i is summed over the years modeled, C_r^{VOM} is the hourly variable operating cost of asset r and Cap_r^h is the capacity of asset r that was operating in hour h.

The fossil fuel plants were constrained to run between a minimum and maximum power. When the output power was selected to drop to zero, the associated cost was added as a "shut-down" cost. When the model selected to restart the plant, a "start-up" cost was added to the objective function as indicated:

$$Start/stop\ cost = \sum_r C_r^{start/shutdown} = \sum_i \sum_r \sum_{h=1}^{8760} \left[C_r^{start}\left(\frac{\$}{start}\right) \times \boldsymbol{Start_r^h} + C_r^{stop}\left(\frac{\$}{stop}\right) \times \boldsymbol{Stop_r^h}\right] \qquad \text{(S3)}$$

Where C_r^{start} is the cost to start asset r and C_r^{stop} is the cost to shut down asset r. $Start_r^h$ and $Start_r^h$ are Booleans to indicate whether asset r was started or shut down in hour h.

The fuel costs were calculated according to

$$Fuel\ cost = \sum_r C_r^{fuel} = \sum_i \sum_r \sum_{h=1}^{8760} C_{rk}\left(\frac{\$}{fuel\ unit}\right) \times \boldsymbol{Cap_r^h}(\boldsymbol{MW}) \times F_r^h(fuel\ unit/MW) \qquad \text{(S4)}$$

Where the summation over i considers the years modeled, C_{rk} is the cost of fuel per unit of fuel k, where the type of fuel varies by asset, and F_r^h is the units of fuel used by asset r per MW powered in hour h.

As described above, curtailment costs were added to the objective function only for plants that were preexisting as in

$$Curtailment\ costs = \sum_s C_s^{curtailment} = \sum_i \sum_s \sum_{h=1}^{8760} C_s^{cur} \left(\frac{\$}{MW}\right) \times \boldsymbol{CapC_s^h(MW)} \qquad \text{(S5)}$$

Where C_s^{cur} is the contracted payment to compensate for curtailment of asset *s* and $CapC_s^h$ is the capacity of asset *s* that was curtailed in hour h.

The hurdle costs were calculated for transmission between the California grid and adjacent grids

$$Hurdle\ cost = \sum_t C_t^{transmission} = \sum_i \sum_t \sum_{h=1}^{8760} \left[C_t^{in} \left(\frac{\$}{MW}\right) \times \boldsymbol{Cap_{t\ in}^h(MW)} + C_t^{out} \left(\frac{\$}{MW}\right) \times \boldsymbol{Cap_{t\ out}^h(MW)}\right] \qquad \text{(S6)}$$

Where *t* indicates the transmission line, $C_t^{in}\left(\frac{\$}{MW}\right)$ is the cost of transmitting a MW of electricity into California for an hour on transmission line *t* and $Cap_{t\ in}^h(MW)$ is the number of MW of electricity that are transmitted into California on line *t* in hour h. Similarly, $C_t^{out}\left(\frac{\$}{MW}\right)$ is the cost of transmitting a MW of electricity out of California for an hour on transmission line *t* and $Cap_{t\ out}^h(MW)$ is the number of MW of electricity that are transmitted out of California on line *t* in hour h.

In general, for the simulations studied in this paper, no penalties were incurred, but for completeness, we describe the penalties that could have been selected by the model in cases for which resources could not be built to be sufficient. The penalties were calculated:

$$Penalty\ cost = \sum_i \sum_z \sum_{h=1}^{8760} \left[C_z^{ul} \left(\frac{\$}{MW}\right) \times \boldsymbol{Cap_{z\ ul}^h(MW)} + C_z^{og} \left(\frac{\$}{MW}\right) \times \boldsymbol{Cap_{z\ og}^h(MW)} + C_z^{ur} \left(\frac{\$}{MW}\right) \times \boldsymbol{Cap_{z\ ur}^h(MW)}\right] \qquad \text{(S7)}$$

Where *z* is the zone, $C_z^{ul}\left(\frac{\$}{MW}\right)$ is the cost of the penalty for unserved load in zone *z* and $Cap_{z\ ul}^h(MW)$ is the unserved load in zone *z* in hour h. Similarly, $C_z^{og}\left(\frac{\$}{MW}\right)$ is the cost of the penalty for overgeneration in zone *z* and $Cap_{z\ og}^h(MW)$ is the overgeneration in zone *z* in hour h. Finally, $C_z^{ur}\left(\frac{\$}{MW}\right)$ is the cost of the penalty for unserved reserve in zone *z* and $Cap_{z\ ur}^h(MW)$ is the unserved reserve in zone *z* in hour h.

The optimization minimizes the cost (objective) function by varying the values for the variables set in bold in equations S1-S7 while constraining all values to align with built capacities and to provide enough electricity to meet demand in each hour plus added reserve.

Table S8. Total generation in 2045 for resources in all of the modeled zones and CAISO and their contribution in meeting the total load in 2045; "other" resources in this table include geothermal, natural gas, coal, nuclear, and biomass

Resource	Energy Generation in All Zones (TWh)	% of Total Load in All Zones	Energy Generation in CAISO (TWh)	% of Total Load in CAISO
Solar	405	43%	376	79%
Wind	81	9%	42	9%
Hydro	149	16%	13	3%
Other	306	32%	41	9%
